# Detection of Drug-Related Problems through a Clinical Decision Support System Used by a Clinical Pharmacy Team

**DOI:** 10.3390/healthcare11060827

**Published:** 2023-03-11

**Authors:** Laurine Robert, Elodie Cuvelier, Chloé Rousselière, Sophie Gautier, Pascal Odou, Jean-Baptiste Beuscart, Bertrand Décaudin

**Affiliations:** 1 CHU Lille, Institut de Pharmacie, F-59000 Lille, France; 2 Univ. Lille, CHU Lille, ULR 7365—GRITA—Groupe de Recherche sur les Formes Injectables et les Technologies Associées, F-59000 Lille, France; 3 Univ. Lille, CHU Lille, INSERM U1171—Centre Régional de Pharmacovigilance, F-59000 Lille, France; 4 Univ. Lille, CHU Lille, ULR 2694—METRICS: Évaluation des Technologies de Santé et des Pratiques Médicales, F-59000 Lille, France

**Keywords:** clinical pharmacist, clinical decision support system (CDSS), drug-related problems

## Abstract

Clinical decision support systems (CDSSs) are intended to detect drug-related problems in real time and might be of value in healthcare institutions with a clinical pharmacy team. The objective was to report the detection of drug-related problems through a CDSS used by an existing clinical pharmacy team over 22 months. It was a retrospective single-center study. A CDSS was integrated in the clinical pharmacy team in July 2019. The investigating clinical pharmacists evaluated the pharmaceutical relevance and physician acceptance rates for critical alerts (i.e., alerts for drug-related problems arising during on-call periods) and noncritical alerts (i.e., prevention alerts arising during the pharmacist’s normal work day) from the CDSS. Of the 3612 alerts triggered, 1554 (43.0%) were critical, and 594 of these 1554 (38.2%) prompted a pharmacist intervention. Of the 2058 (57.0%) noncritical alerts, 475 of these 2058 (23.1%) prompted a pharmacist intervention. About two-thirds of the total pharmacist interventions (PI) were accepted by physicians; the proportion was 71.2% for critical alerts (i.e., 19 critical alerts per month vs. 12.5 noncritical alerts per month). Some alerts were pharmaceutically irrelevant—mainly due to poor performance by the CDSS. Our results suggest that a CDSS is a useful decision-support tool for a hospital pharmacist’s clinical practice. It can help to prioritize drug-related problems by distinguishing critical and noncritical alerts. However, building an appropriate organizational structure around the CDSS is important for correct operation.

## 1. Introduction

In recent years, clinical pharmacists have been in great demand in hospitals and their range of activities has broadened considerably. Clinical pharmacists enhance quality patient care by making medication use safer and more effective, in collaboration with physicians and nurses. Prescription review is one of the clinical pharmacist’s primary tasks; this involvement is known to reduce the number of prescription errors leading to adverse drug events [1]. As part of this process, the detection of drug interactions and drug contraindications has been facilitated by the emergence of computerized physician order entry (CPOE) in hospitals [2]. The presence of clinical pharmacist in wards using a CPOE is associated with high pharmacist intervention (PI) acceptance rates [3]. Furthermore, collaborative working with other health professionals increases the quality of patient care [4,5,6,7]. Clinical pharmacy activities enhance the safety, appropriateness and efficiency of drug therapy through medication reconciliation and medication review [8,9,10]. Nevertheless, adverse drug reactions are still often encountered in hospitals, and some are preventable; they extend the length of hospital stay and have a negative economic impact [11,12,13,14].

The introduction of clinical decision support systems (CDSSs) has been associated with positive clinical and economic impacts [9,15]. CDSSs promote specific drug safety alerts for medication monitoring and can be combined with CPOE to increase the healthcare provider’s performance [7]. A recent review highlighted the role of CDSSs in improving high-quality medication use and enhancing the quality of care [16]. In fact, CDSSs are real-time tools for detecting at-risk situations and thus decreasing medical errors and adverse drug events [17]. However, a large body of literature data has highlighted barriers to use for CDSSs, such as alert fatigue and the overriding of relevant alerts [18,19]. It has been suggested that pharmacist management of a CDSS can decrease the number of irrelevant alerts transmitted to physicians [20].

Given an increase in clinical pharmacy activities and the number of inpatient beds with computerized data, Lille University Medical Center (Lille, France) sought to prioritize prescriptions by alerting clinical pharmacists to potential drug-related problems. Hence, a CDSS was implemented. The objective of the present study was to report the detection of drug-related problems through a CDSS used by an existing clinical pharmacy team.

## 2. Materials and Methods

### 2.1. Ethical Approval

In line with the French legislation on retrospective studies of clinical practice, the study protocol was approved by a hospital committee with competency for research not requiring approval by an institutional review board (Lille University Medical Center; reference: ID1080).

### 2.2. Study Design

This retrospective, observational study was conducted at Lille University Medical Center over a 22-month period (1 July 2019 to 30 April 2021). The study data covered 1348 inpatient beds on nonsurgical wards (e.g., pulmonology, cardiology, geriatrics and neurology wards, etc.), surgical wards and psychiatric wards. The clinical pharmacy team included 18 senior pharmacists and 8 pharmacy residents. Each clinical pharmacist was responsible for specific wards. The clinical pharmacists conducted patient interviews about specific medications, performed inpatient and outpatient medication reconciliations, and analyzed patient prescriptions (i.e., medication reviews). These activities sometimes prompted the pharmacist to issue a pharmacist intervention (PI), which was communicated to the prescribing physician through the local CPOE or by phone. In July 2019, a CDSS (PharmaClass^®^, Keenturtle, Paris, France) was integrated into the clinical pharmacy team’s everyday workflow. The CDSS did not send alerts that interrupted the pharmacist’s work; the pharmacist had to connect to the system and consult the alerts. This CDSS is separate from the hospital’s joint CPOE and electronic health record software (Sillage^®^, SIB, Rennes, France).

### 2.3. Creation and Optimization of Clinical Rules

The CDSS was powered by the integration of rules into the system. Clinical rules were developed according to a standardized procedure. Firstly, the rules were suggested and worked on by pharmacists upstream from the project team (composed of three clinical pharmacists in charge of the management and the development of the CDSS). The rules were based on expert recommendations, national and international guidelines, summaries of product characteristics, or clinical practice. Next, the rules were submitted to the clinical pharmacy team and the project committee (composed of pharmacists, physicians, pharmacologists, and medical biologists) for validation. This dual, multidisciplinary effects validation process enabled a clear definition of the criteria triggering the rule, the rule’s level of criticality, the suggested PI, and the course of action, as described below:

- Rule criteria were based on data collected by the CDSS: laboratory results (e.g., a threshold of 5 mM for blood potassium), drug prescriptions, drug dose levels (e.g., the prescription of more than 1000 mg of metformin three times a day) and the patient’s administrative data (e.g., age over 75).
- Criticality of the alert was based on the validated French classification of clinical, health, economic, and organizational impacts of PIs developed by the French Society of Clinical Pharmacy (Société Française de Pharmacie Clinique (SFPC)) (see Suppl. Data Table S1) [21]. Criticality is based on the addition of these various weighted impacts. The establishment of criticality rules allowed us to distinguish between critical and noncritical alerts. Critical alerts were defined as those with a criticality score of 7 or more (e.g., maintaining an antivitamin K with an INR ≥ 6). This criticality threshold of 7 corresponds to a threshold where rapid intervention was deemed necessary according to the project committee and the clinical pharmacy team. Noncritical alerts were defined as those with a criticality score below 7 (e.g., prescription of hypoglycemic sulfonamide among elderly patients).
- Pharmacist interventions (PIs) and the course of action were proposed for each rule by the project committee and the clinical pharmacy team, based on guidelines or clinical expertise. The PI could recommend definitive discontinuation, temporary discontinuation or the modification of drug treatment, patient monitoring, or laboratory assays. The course of action was also proposed in order to provide to the clinical pharmacy team the process of what to do when an alert was triggered. This course of action was validated by the entire clinical pharmacy team.

The rules were classified according to the associated problems, based on the SFPC’s ActIP^®^ classification: an adverse drug reaction (four rules), a drug interaction (nine rules), an inappropriate administration route/mode (three rules), monitoring (nine rules), a noncompliance with guidelines/contraindication (28 rules), a nonindicated drug (five rules), overdosing (27 rules), underdosing (one rule), and an untreated indication (three rules). The rules were classified independently by two investigators (L.R. and E.C.), and the results were pooled. Details are given in Suppl. Data Table S2.

During the use of the CDSS, the rules were improved by taking account of (i) feedback from the pharmacists using CDSS and (ii) changes over time in the available data. In fact, not all of the information available in electronic health records was available for rule creation in the CDSS: we did not have access to data on vital signs or (before October 2020) drug dose levels.

There were 34 clinical rules in July 2019 and 89 in April 2021. Details of the changes in clinical rules over the study period are given in Suppl. Data Figure S1.

### 2.4. Organization of the Clinical Pharmacy Team following Implementation of the CDSS

After the CDSS was integrated into the clinical pharmacy team, the pharmacists’ routine practice changed slightly. All pharmacists and pharmacy residents were trained in and continuously updated about this activity. For non-critical alerts (i.e., criticality score below 7), each clinical pharmacist/pharmacy resident had to analyze alerts from his/her referring medical wards on a daily basis during his/her normal workday hours. For critical alerts (i.e., criticality score of 7 or more), a member of the clinical pharmacy team was on call (from 8:30 a.m. to 6:30 p.m. on weekdays) to analyze critical alerts (triggered by 52 clinical rules). Alerts triggered outside these periods were seen the following day or on Monday (if the alert triggered during the weekend). When on call, the clinical pharmacist/pharmacy resident analyzed all critical alerts from all wards covered by the CDSS (not only the wards for which he/she was responsible), and contact, if the alert was technically valid, the ward’s referring pharmacist to inform on the alert triggered. This referring pharmacist was the most appropriate to conduct the PIs, as he/she was more familiar with the ward’s habits and physicians.

Each CDSS alert analyzed was entered by the pharmacist on a Excel^®^ spreadsheet.

### 2.5. Analysis of Alerts/Monitoring of Indicators

Each alert from the CDSS was analyzed with regard to the analysis date, identification number, medical ward, rules triggered, criticality of the rule, and the pharmacist’s evaluation. This evaluation was standardized and consisted of three main steps:

- The alert’s technical validity: an alert was deemed to be valid when criteria required to trigger the rule were met (administrative, laboratory and treatment-related data). If the terms of the rule were met, the alert was qualified as “technically valid”. Technically invalid alerts were alerts that did not meet exactly the criteria of the rule, due to data integration issues (long reception time, lack of data integration …). For example, it was alerts that detected at-risk situations in which medication was already suspended or discontinued.
- The alert’s pharmaceutical relevance: only a technically valid alert could prompt the clinical pharmacist to issue a PI. However, a PI was issued only if the clinical pharmacist considered that the prescription or change was relevant for medical care. If a PI was issued, the problem and the action suggested by the pharmacist were noted. The transmission channel for the PI, the type of problem and action suggested following an alert were based on the SFPC’s classification [22,23]. An alert prompting a PI is referred to hereafter as “pharmaceutically relevant”.
- Physician acceptance of a PI: a PI (a suggestion for treatment or monitoring by the pharmacist) could be accepted or not by the prescribing physician.

**Figure 1:**
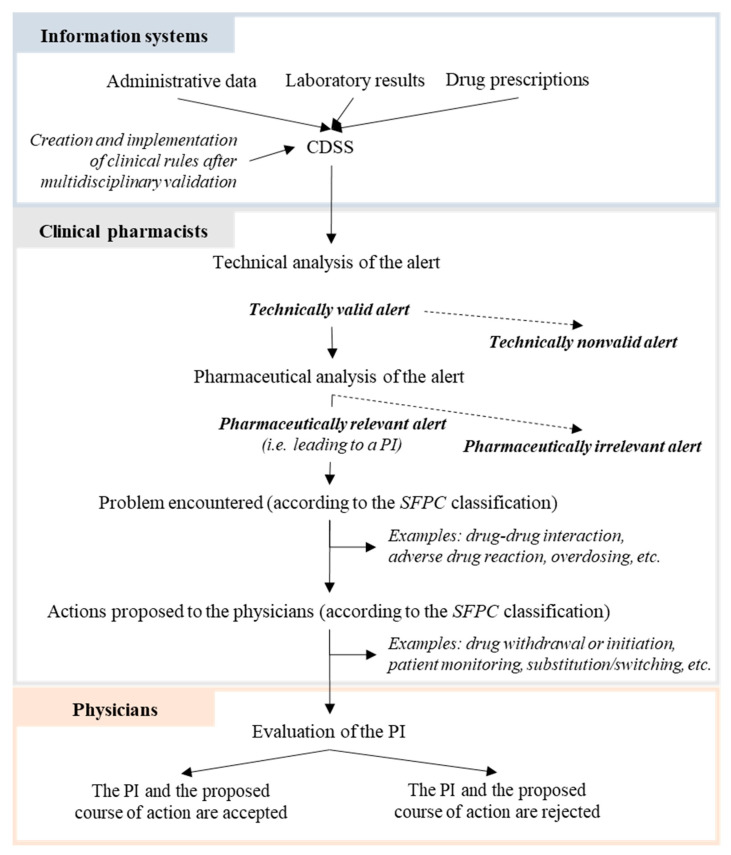
Analysis of CDSS alerts by the clinical pharmacists. CDSS: clinical decision support system; PI: pharmacist intervention; SFPC: French Society of Clinical Pharmacy.

We recorded the reasons for technically invalid alerts and pharmaceutically irrelevant alerts. However, we did not know why a physician decided to refuse a PI. The prescription analysis process with the CDSS is shown in Figure 1.

The pharmaceutically relevant rate was defined as the number of alerts leading to a PI as a percentage of the total number of technically valid alerts. The PI acceptance rate was defined as the number of PIs accepted by the physician as a percentage of the total number of PIs.

### 2.6. Data Sources and Extraction

The study data encompassed the information entered by the clinical pharmacist in the Excel^®^ 2013 (Microsoft Corporation, Redmond, WA, USA) spreadsheet (including information on the alert and its rating), the data in the CPOE (the PIs issued by the pharmacist), and the data in the CDSS (including the criteria that triggered the alert and the pharmacist’s rating of the alert). By cross-referencing these data, we extracted the number of alerts triggered during the study period, the number of technically valid alerts, the number of pharmaceutically relevant alerts, and the PI acceptance rate.

### 2.7. Statistical Analysis

All statistical analyses were performed using R Studio^®^ software (version 3.3.3, R Foundation for Statistical Computing, Vienna, Austria) [24]. Qualitative variables were expressed as the frequency (percentage). We used a chi-squared test to compare categorical variables. The threshold for statistical significance was set to *p* < 0.05.

## 3. Results

### 3.1. Analysis of Critical vs. Noncritical Alerts

**Figure 2:**
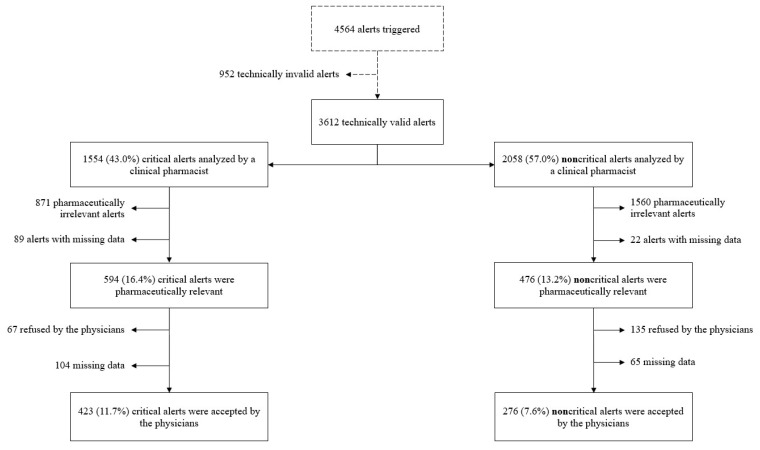
Flow chart of the analysis of critical and noncritical alerts triggered by the CDSS during the 22-month study (the data are reported as proportions of the 3612 technically valid alerts).

During the study period, 4564 alerts were triggered by the CDSS (i.e., a mean of 208 alerts per month). On the 3612 technically valid alerts, 1554 (a mean of 71 alerts per month) were critical alerts and had been analyzed during an on-call period. A clinical pharmacist issued a PI for 594/1554 (38.2%) of these technical valid critical alerts (16.4% of the whole alerts, Figure 1). The PI acceptance rate was 71.2% (423 over 594), corresponding to an average of 19 alerts per month (11.7% of the whole alerts, Figure 1). At the same time, noncritical alerts led to a PI in 476/2058 (23.1%) cases, of which 276 (58.0%) were accepted by the physicians (i.e., a mean of 12.5 alerts per month, corresponding to 7.6% of whole alert, Figure 1). The acceptance rate was significantly higher for critical alerts than for noncritical alerts (*p* < 0.001). Details of the alert analysis are given in Figure 2. 

The pharmaceutical relevance rate was 28.5% before inclusion of the drug dose level in the CDSS (i.e., before October 2020) and 32.0% afterwards; this increase was significant (*p* = 0.03). The acceptance rate also increased significantly, from 61.5% before inclusion of the drug dose level to 72.2% afterwards (*p* < 0.001).

### 3.2. Characteristics of the PIs

During the on-call period, the problems that mainly led to a PI were noncompliance with guidelines or a contraindication (38.2%), and drug interaction (27.8%). Most of the actions initiated following a PI corresponded to definitive discontinuation of the drug: 81.5% of these PIs were accepted. The problems that mainly led to a PI during routine work were noncompliance/contraindication (46.6%) and overdosing (33.2%). Most of the PIs led to drug substitution/switching (34.7%) and adjustment of the dose level (27.5%), but the PI acceptance rates were lower (52.1% and 66.4%, respectively). The alerts and PIs are summarized in Suppl. Data Table S3.

During the study, the PIs for noncritical alerts were mainly issued through the CPOE (53.6% of all pharmaceutically relevant PI), with 16.6% issued on the ward and 9.7% issued by phone. For critical alerts, PIs were less likely to be issued through the CPOE (45.3%; *p* = 0.007) and more likely to be issued by phone (26.9%; *p* < 0.001).

The pharmaceutical irrelevance rate was high: 56.0% for critical alerts and 75.8% for noncritical alerts. The most common reason was poor performance of the CDSS, including the need to refine rules when more data could be integrated into the CDSS, or a need for faster data integration because the physician could have changed the prescription before the CDSS issued an alert. The other causes of irrelevant PIs were essentially because the pharmacist preferred to monitor the progression of the patient’s condition before suggesting a course of action, or PIs are not issued in a given ward according to the physicians’ prescribing habits (ex: for the rule “switch from enoxaparin to tinzaparin”, the PI was always refused by surgery ward because the requirement for heparin is frequently re-evaluated). Details of pharmaceutical irrelevance are given in Table 1.

### 3.3. Categories of Rules Triggered

For technically valid alerts, the main rule triggered for noncritical alerts was the use of a curative dose of enoxaparin rather than tinzaparin (478 alerts over the 22 months). For critical alerts, the main rule was the presence of hyperkalemia and the maintenance of potassium intake (259 alerts over the 22 months). With regard to categories of rules, rules on noncompliance with guidelines/contraindication were the most frequent, and the proportion of PIs increased with the criticality of the alert. The drug interaction category was less frequent but was responsible for many of the PIs (49.6%). Lastly, rules on monitoring and overdosing led to few PIs but were accepted by physicians in 73.0% and 72.2% of cases, respectively. The PIs and their acceptance were related to the criticality of the alerts, which in turn varied according to the type of rule. The pharmaceutical relevance of potential adverse drug reactions was greater for critical alerts (44.8%) than for all alerts (28.6%). Among PIs corresponding to critical alerts, those related to overdosing had a high acceptance rate (78.5%). The same was true for PIs concerning noncompliance/contraindications and drug interactions, which were accepted by the physician in 73.1% and 72.7% of cases, respectively. Among noncritical alerts, the “inappropriate administration route/mode” rule had the higher pharmaceutical relevance rate and PI acceptance rate (90.9% and 90.0% respectively). Rules on monitoring, overdosing, and a nonindicated drug led to fewer PIs but were often accepted by the physician (73.0%, 67.1% and 61.5% respectively). Details of the analysis of categories of rules applied to technically valid alerts are given in Table 2.

## 4. Discussion

Our results showed that the CDSSs lead to the generation of PIs in 29.6% of the alerts and that are accepted by the prescribing physicians in 65.3% of the cases. We applied several strategies, including the classification of the rules by criticality and the analysis of critical alerts during on-call periods only. The pharmacists were free to consult (or not) alerts generated outside the on-call periods.

Using the CDSS for the clinical pharmacist’s daily work generated 3612 technically valid alerts. Although less than half were critical alerts (43.0%), the latter were more likely to lead to a PI and were often accepted by physicians. Although noncritical alerts accounted for 57.0% of the total, few led to a PI. Changes in the prescription accounted for about 19 critical alerts and 12.5 noncritical alerts per month.

CDSSs are useful tools that can increase the number of PIs, thanks to their interoperability with other health system flows [15]. However, further development is required to avoid alert fatigue and incorrect overriding due to nonspecific alerts [25]. In our study, about 20% of alerts were technically invalid and required technical investigation. This high technical invalidity rate might lead to alert fatigue in the pharmacist. A work collaboration is needed with the editors of the CDSS to improve data integration, interoperability with others’ systems, and performance of the tool in order to remedy these technically invalid alerts. The CDSS’s ability to generate only appropriate alerts is limited by ergonomic, organizational, and technical barriers (e.g., data reception load, redundancy of alerts) [25]. Some researchers have suggested decreasing alert fatigue by sorting alerts by severity, sending the alert to the pharmacist, or recording the reason for overriding the alert [26,27]. These suggestions were applied to our CDSS, although technical and ergonomic improvements are still required. Improving and creating the interoperability of data flows (e.g., vital signs) to be more specific could help to make CDSSs more relevant. The involvement of clinical pharmacists is essential for the integration of alerts into the CDSS and the reduction of inappropriate alerts [4]. In fact, clinical pharmacists translate the data from the automated alert into information for physicians. Moreover, a lot of work is needed to increase alert specificity and to avoid the over-alerting encountered with irrelevant rules [28]. One way of increasing alert specificity might be the use of machine learning to identify drug-related problems (adverse drug events or treatment errors). Machine learning might increase accuracy and decrease the false positive alert rate [29,30,31].

Another key finding was the low proportion of pharmaceutically relevant alerts. Only 16.4% of critical technical alerts and 13.2% of the noncritical technical alerts prompted a PI. The creation of an on-call roster for reviewing critical alerts in real time highlighted (i) the value of measuring criticality in PI transmission and acceptance, and (ii) the fact that an appropriate organizational structure around the CDSS is essential. Our results show that the PI acceptance rate was higher for alerts analyzed during the on-call period than for alerts analyzed during the clinical pharmacist’s daily work. However, this new activity had to be performed in addition to the clinical pharmacist’s existing activities and so increased the workload. Of the technically valid alerts, 1543 were judged to be irrelevant by the clinical pharmacist because of the CDSS’s poor specificity. Rommers et al. found that 90.0% of alerts were irrelevant, according to the pharmacists [32]. The main causes were very similar to those observed in our study: a delay between the alert and the prescription change, irrelevant alerts in certain wards, and redundant alerts. In the present study, alerts caused by poor performance of the CDSS (n = 1543) were cumulated with technically invalid alerts (n = 952); these alerts accounted for 54.7% of the CDSS’s irrelevant alerts. The inclusion of the drug dose level in October 2020 was associated with a slight reduction in the number of pharmaceutically irrelevant alerts, but work still has to be done on data integration and system interoperability if false positive alerts are to be avoided. Assessing pharmaceutical irrelevance makes it possible to highlight changes over time in the interface; this can help to improve the system and refine the rules to be more relevant.

Some alerts will always be pharmaceutically irrelevant because they will not fit with the ward’s habits. A lot of clinical rules are not specific to a given ward and so the associated alerts might be overridden more frequently. However, the objective of the CDSS’s implementation was to standardize the course of action to be taken following an alert. Some wards (e.g., surgical wards) may have different clinical practices and different care objectives, leading to the systematic overriding of alerts. An analysis of the PIs and their acceptance by the medical team in different wards could be an interesting focus.

In our study, half of the PIs were transmitted through the CPOE; this depended on the criticality of the alert, and the habits of the ward and its physicians. Lagreula et al. compared pharmaceutical validation in the central pharmacy and on the ward. More PIs were issued on the ward (13.3%, vs. 2.9% in the central pharmacy) and the on-ward PIs had a higher acceptance rate (84%, vs. 65% in the central pharmacy) [33]. Transmission of a PI on the ward whenever possible might increase the PI acceptance rate.

Our study highlighted the main categories triggered during the study period, based on critical and noncritical alerts. The main category was noncompliance/contraindication, and about one-third were pharmaceutically relevant. Critical alerts about drug interaction were the main category leading to an accepted PI. Our results were in line with the findings of Quintens et al.: the alerts mainly concerned drug interactions (50%) and drug use in patients with renal insufficiency (25%) [34]. For noncritical alerts, the main pharmaceutically relevant category with accepted PIs related to nonindicated drugs. This finding raises the question of whether or not the CDSS is valuable. This question should be discussed in collaboration with the pharmacy team that will be using the system. Do they want to be alerted only to situations in which a PI will be issued (i.e., affecting hospital care) or also to prevention-related situations that do not necessarily lead to a PI (i.e., affecting primary care mainly)?

Implementing such systems needed reliability and validation. CDSSs are mainly complementary and alert pharmacist on at-risk situations, and at this time of the development in our institution, these tools do not block patient administration. Indeed, CDSSs are not yet sufficiently reliable and blocking administration may not be relevant and could lead to task interruptions. A CDSS completed the “traditional” process of clinical pharmacist to globally reduce drug-related problems. Its use was complementary to the pharmaceutical analysis and the daily use allowed us to improve the tool by feedback to re-evaluate the clinical rules and to improve the tool in connection with the technical team of the editor of the system. Sharing clinical rules to implement in the CDSS may be interesting to be done to create a common basis for work. This work is in progress but some barriers prevent direct diffusion and implementation into another hospital (e.g., data availability, software issue, intellectual property).

Our study had a number of strengths. Firstly, the study period was quite long (22 months). Secondly, we analyzed a large quantity of computerized data from a teaching hospital. Thirdly, the study covered a large specialized clinical pharmacy team, with a standardized analytical method (e.g., an on-call roster, rule criticality, and alert traceability).

The study also had some limitations. Firstly, we did not compare clinical practice in the presence vs. absence of a CDSS. Once a support tool has been implemented, it is difficult to set up a comparative study. Better designed, longer-term studies are needed for a more reliable evidence base on the CDSS’s ability to detect drug-related problems. Secondly, the alert analyses were reported on a separate spreadsheet, which added an additional constraint for the clinical pharmacists in their daily practice. This might have contributed to the high proportion of missing data, and so our results might be underestimated. In fact, traceability of alerts could sometimes be complicated because it was time-consuming, because of the ergonomy of the tool, and especially for the complex follow-up of PIs. Lastly, there was a risk of interpretation bias because alerts analyzed during routine practice were not independently validated by two pharmacists. However, this approach reflected the clinical reality.

## 5. Conclusions

The results of the present study showed that a CDSS is a promising tool for long-term use but that a large amount of work is required to operate the system. Distinguishing between critical and noncritical alerts is essential for detecting imminent risk situations during the activity of a well-established clinical pharmacy team. The criticality of an alert appears to influence the pharmacist’s behavior and the PI acceptance rate by the physicians. An analysis of critical alerts helps to prioritize pharmaceutical analysis activities, especially when the ward’s clinical pharmacist is performing other activities (medication reconciliation, pharmaceutical reviews, etc.) The analysis also enables the identification of medication-related problems and high-risk situations, leading to a PI in about 40% of cases and a 70% PI acceptance rate. Implementation of a CDSS creates a new activity for clinical pharmacists—notably the implementation of an on-call roster for detecting critical alerts; this highlighted the CDSS’s contribution to the real-time detection of drug-related problems in hospital. Nevertheless, the CDSS generated extra work, especially when the alerts were irrelevant. In principle, CDSSs are very useful but must be adapted to suit a hospital pharmacist’s clinical practice.

## Figures and Tables

**Table 1:** Reasons for the pharmaceutical irrelevance of critical and noncritical alerts, according to the clinical pharmacist.

Reason	Critical Alerts n = 871	Noncritical Alerts n = 1560
Poor performance by the CDSS, n (%)	539 (61.9%)	1004 (64.4%)
Pharmacist’s decision to monitor the patient, n (%)	136 (15.6%)	254 (16.3%)
Irrelevant alert for the ward in question, n (%)	70 (8.0%)	208 (13.3%)
Other, n (%)	122 (14.0%)	60 (3.8%)
Missing data, n (%)	4 (0.5%)	34 (2.2%)

CDSS: clinical decision support system.

**Table 2:** Analysis of the categories of rules for all alerts, critical alerts (issued during on-call periods) and noncritical alerts.

Categories of Rule	All Alerts			Critical Alerts			Noncritical Alerts		
	Tech. Valid	Pharm. Relevant n (%) *	PI Accepted n (%) **	Tech. Valid	Pharm. Relevant n (%) *	PI Accepted n (%) **	Tech. Valid	Pharm. Relevant n (%) *	PI Accepted n (%) **
Adverse drug reaction	84	24 (28.6%)	11 (45.8%)	29	13 (44.8%)	6 (46.2%)	55	11 (20.0%)	5 (45.5%)
Untreated indication	74	20 (27.0%)	11 (55.0%)	32	9 (28.1%)	5 (55.6%)	42	11 (26.2%)	6 (54.5%)
Drug interaction	341	169 (49.6%)	122 (72.2%)	332	165 (49.7%)	120 (72.7%)	9	4 (44.4%)	2 (50.0%)
Nonindicated drug	149	46 (30.9%)	27 (58.7%)	123	33 (26.8%)	19 (57.6%)	26	13 (50.0%)	8 (61.5%)
Monitoring	181	37 (20.4%)	27 (73.0%)	0	0	0	181	37 (20.4%)	27 (73.0%)
Noncompliance with guidelines/contraindication	1474	449 (30.5%)	270 (60.1%)	554	227 (41.0%)	166 (73.1%)	920	222 (24.1%)	104 (46.8%)
Underdosing	128	23 (18.0%)	11 (47.8%)	128	23 (20.0%)	11 (47.8%)	0	0	0
Overdosing	1156	279 (24.1%)	201 (72.0%)	353	121 (34.3%)	95 (78.5%)	803	158 (19.7%)	106 (67.1%)
Inappropriate administration route/mode	25	23 (92.0%)	19 (82.6%)	3	3 (100.0%)	1 (33.3%)	22	20 (90.9%)	18 (90.0%)

PI: pharmacist intervention; Pharm: pharmaceutically; Tech: technically. * Proportion are calculated on the number of technically valid alert triggered. ** Proportions are calculated on the number of pharmaceutically relevant alert.

## Data Availability

Not applicable.

## Supplementary Materials

The following supporting information can be downloaded at: https://www.mdpi.com/article/10.3390/healthcare11060827/s1, Table S1: Definition of alert criticality, according to the SFPC classification; Figure S1: The study timeline: date of rule creation, and change over time in the number of active rules in the CDSS and the number of inpatients’ beds during the study; Table S2: Details of rules (category, name, and criticality) and number of technically valid alerts triggered during the study; Table S3: Actions suggested by clinical pharmacists and their acceptance by physicians during the study period for critical and noncritical alerts.
