# Supplementary material for: Detection of Drug-Related Problems through a Clinical Decision Support System Used by a Clinical Pharmacy Team
Source: Healthcare (Basel). 2023 Mar 11;11(6):827. doi: 10.3390/healthcare11060827 (PMC10048130; doi:10.3390/healthcare11060827)
Supplement: Supplementary file 1 [file healthcare-11-00827-s001.zip › healthcare-2202935-supplementary.pdf]

Detection of drug-related problems through a clinical decision support system used by a clinical pharmacy team

<u>Supplementary materials:</u>

Table S1: Definition of alert criticality, according to the SFPC classification

Figure S1: The study timeline: date of rule creation, and change over time in the number of active rules in the CDSS and the number of inpatients beds during the study.

Table S2: Details of rules (category, name and criticality) and number of technically valid alerts triggered during the study.

Table S3: Actions suggested by clinical pharmacists and their acceptance by physicians during the study period for critical and noncritical alerts.

Supplementary information:

Table S1: Definition of alert criticality, according to the SFPC classification.

Clinical impact						
**Weighting coefficient**	2					
**Nature of the impact**	harmful	null	minor	medium	important	vital
**CLEO score**	-1	0	1	2	3	4
**Criticality score**	0	0	2	4	6	8

Organizational impact			
**Weighting coefficient**	1		
**Nature of the impact**	unfavorable	null	favorable
**CLEO score**	-1	0	1
**Criticality score**	0	0	1

Health economic impact			
**Weighting coefficient**	1		
**Nature of the impact**	cost increase	no change	cost reduction
**CLEO score**	-1	0	1
**Criticality score**	0	0	1

The total score is the sum of the three CLEO scores (clinical impact, organizational impact and health economic impact), each of which is rated from 0 to 10.

Figure S1: The study timeline: date of rule creation, and change over time in the number of active rules in the CDSS and the number of inpatients beds during the study.

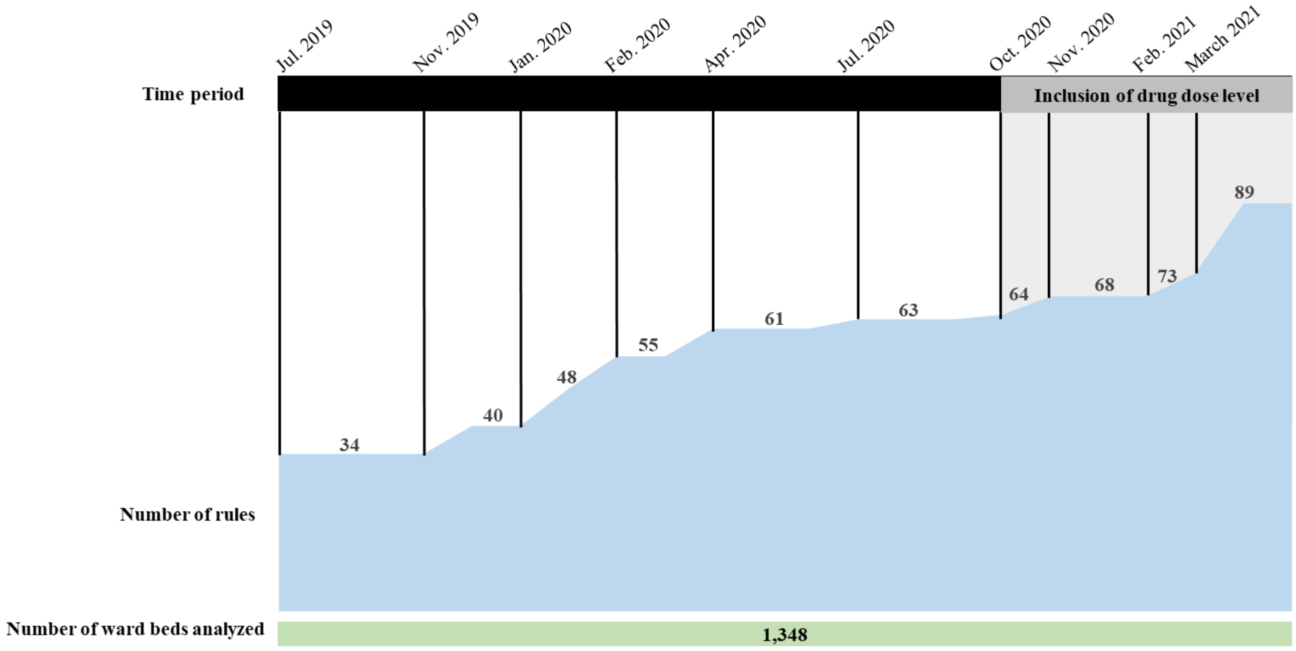

Table S2: Details of rules (category, name and criticality) and number of technically valid alerts triggered during the study.

Rule category	Rule name	Criticality score	Number of technically valid alerts	Number of pharmaceuti-cally relevant alert	Number of medically relevant alert
Adverse reaction	Clozapine and a very low CBC	8	5	3	1
Adverse reaction	Colchicine and an antidiarrheal agent	8	19	9	4
Adverse reaction	Hyponatremic drug and hyponatremia	5	55	11	5
Adverse reaction	Statin, daptomycin and a high creatine phosphokinase level	8	5	1	1
Nonindicated drug	Co-prescription of inhaled beta 2 agonists	5	10	8	4
Nonindicated drug	Co-prescription of inhaled corticoids	5	3	2	2
Nonindicated drug	Co-prescription of inhaled anticholinergics	5	2	1	1
Nonindicated drug	Premixed and low insulin	7	123	33	19
Nonindicated drug	Co-prescription of an antivitamin K and heparin, with three INRs between 2 and 3	6	11	2	1
Drug-drug interaction	Colchicine and combination with contraindicated antibiotics	9	6	3	3
Drug-drug interaction	Digoxin and intravenous calcium	8	0	0	0
Drug-drug interaction	Co-prescription of a glinide and sulfamide in a patient with CKD	5	9	4	2
Drug-drug interaction	Lopinavir/ritonavir and contraindicated drugs	7	4	1	0
Drug-drug interaction	Linezolid and selective serotonin reuptake inhibitors	7	87	49	35
Drug-drug interaction	Linezolid and tramadol	7	168	99	80
Drug-drug interaction	Metformin and iodinate contrast agent	9	0	0	0
Drug-drug interaction	Methotrexate and antibiotic, proton pump inhibitors or sulfamide	7	59	8	5
Drug-drug interaction	Hydroxychloroquin and contraindicated drugs	7	8	5	4
Inappropriate administration route/mode	Anticoagulant and intramuscular route	5	9	8	7
Inappropriate administration route/mode	Tiotropium bromure monohydrate and dosing regimen	4	13	12	10
Inappropriate administration route/mode	Tacrolimus and dosing regimen	7	3	3	1
Monitoring	Clozapine and moderately low CBC	5	5	2	1
Monitoring	Digoxin and moderate hypokalemia	4	1	0	0

Monitoring	Digoxin and severe hypokalemia	7	0	0	0
Monitoring	AKI, old age, and hyperkalemia	6	20	7	5
Monitoring	Linezolid and the absence of a complete blood count in the previous week	4	0	0	0
Monitoring	Prevention of hyperkalemia and potassium intake	3	85	15	12
Monitoring	Co-prescription of an antivitamin K and heparin, with two INRs between 2 and 3	6	0	0	0
Monitoring	Statin, daptomycin but no CPK assay data in the previous week	5	0	0	0
Monitoring	Vancomycin prescription for less than 3 days	0	70	12	7
Noncompliance/ contraindication	DOA and severe CKD	7	26	7	5
Noncompliance/ contraindication	NSAID among elderly	4	11	1	0
Noncompliance/ contraindication	Calciparin without CKD or AKI	6	353	121	69
Noncompliance/ contraindication	Dabigatran and severe CKD	7	1	0	0
Noncompliance/ contraindication	Digoxin among elderly	6	8	5	1
Noncompliance/ contraindication	AKI and nephrotoxic drug: aminoside	7	0	0	0
Noncompliance/ contraindication	AKI and nephrotoxic drug: antivirals	7	1	0	0
Noncompliance/ contraindication	AKI and nephrotoxic drug: immunosuppressants	7	1	0	0
Noncompliance/ contraindication	AKI and nephrotoxic drug: vancomycin	7	1	0	0
Noncompliance/ contraindication	AKI and nephrotoxic drug: antifungal	7	0	0	0
Noncompliance/ contraindication	AKI and drug re-evaluation: NSAID and digoxin	7	2	1	1
Noncompliance/ contraindication	AKI and drug re-evaluation: anticoagulant	7	27	4	3
Noncompliance/ contraindication	AKI and drug re-evaluation: antifungal	7	3	0	0
Noncompliance/ contraindication	AKI and drug re-evaluation: various antibiotic	7	2	0	0
Noncompliance/ contraindication	AKI and drug re-evaluation: beta-blocker	7	19	1	1
Noncompliance/ contraindication	AKI and drug re-evaluation: cephalosporin	7	4	3	2
Noncompliance/ contraindication	AKI and drug re-evaluation: antidiabetic	7	4	1	0
Noncompliance/ contraindication	AKI and drug re-evaluation: various drug	7	10	5	4
Noncompliance/ contraindication	AKI and drug re-evaluation: fluoroquinolone	7	1	0	0
Noncompliance/ contraindication	AKI and drug re-evaluation: cardiology drug	7	31	5	4
Noncompliance/ contraindication	AKI and drug re-evaluation: penicillin	7	6	0	0

Noncompliance/ contraindication	Switch from enoxaparin to tinzaparin	2	478	79	28
Noncompliance/ contraindication	Metformin and severe CKD	10	92	48	31
Noncompliance/ contraindication	Metformin and AKI	10	68	40	33
Noncompliance/ contraindication	ARB or ACEi and AKI	7	246	105	76
Noncompliance/ contraindication	Hypoglycemic sulfonamide and elderly	4	70	19	6
Noncompliance/ contraindication	Hypoglycemic sulfonamide and severe CKD	7	8	4	4
Noncompliance/ contraindication	Prescription of a diuretic, NSAID and ACEi or ARB and AKI	8	1	1	1
Overdosing	Dabigatran adjustment 001	4	30	2	2
Overdosing	Dabigatran adjustment 002	4	5	2	0
Overdosing	Dabigatran adjustment 003	4	16	5	2
Overdosing	Rivaroxaban adjustment to renal function	4	32	13	7
Overdosing	Allopurinol and severe CKD	4	17	4	3
Overdosing	Apixaban half dose adjustment	4	30	6	4
Overdosing	Antivitamin K and INR between 3-4	4	131	18	12
Overdosing	Antivitamin K and INR between 4-6	5	59	13	13
Overdosing	Antivitamin K and INR ≥ 6	8	8	3	2
Overdosing	Antivitamin K and INR between 6-9	8	30	18	15
Overdosing	Antivitamin K and INR ≥ 10	10	3	2	2
Overdosing	Daily bisphosphonate	7	12	4	2
Overdosing	Colchicine and maximum daily dose	8	16	9	6
Overdosing	Colchicine and maximum dose level per dose	8	5	4	3
Overdosing	Digoxin and severe CKD	9	7	4	3
Overdosing	Control of anticoagulant doses	10	4	0	0
Overdosing	Hyperkalemia and potassium intake	8	259	79	57
Overdosing	Premixed insulin prescribed 3 times a day	7	1	0	0
Overdosing	Metformin and mild CKD	4	58	7	6
Overdosing	Metformin and moderate CKD	6	150	26	20
Overdosing	Daily prescription of methotrexate	10	3	0	0
Overdosing	Oseltamivir and severe CKD	7	5	0	0
Overdosing	Saxagliptin and CKD	4	4	3	1
Overdosing	Sitagliptin and mild to moderate CKD	4	158	32	18
Overdosing	Sitagliptin and severe CKD	6	23	15	12
Overdosing	Prescription of synacthen for more than 2 days	8	0	0	0
Overdosing	Vildagliptin and CKD	4	90	10	9
Underdosing	Apixaban sub-therapeutic dose	7	128	23	11

Untreated indication	Antivitamin K, INR below target in the absence of heparin	6	35	10	5
Untreated indication	INR ≥ 6 and no vitamin K	8	32	9	4
Untreated indication	Methotrexate in the absence of folic acid	6	7	1	1

ACEi: angiotensin converting enzyme inhibitor; AKI: acute kidney injury; ARB: angiotensin receptor blocker; CBC: complete blood count; CKD: chronic kidney disease; DOA: direct oral anticoagulant; NSAID: non-steroidal anti-inflammatory drug

Table S3: Actions suggested by clinical pharmacists and their acceptance by physicians during the study period for critical and noncritical alerts

Actions suggested	Critical alerts (N= 594)		Noncritical alerts (N= 476)	
	**Pharm. relevant n (%)**	**Acceptance n (%)***	**Pharm. relevant n (%)**	**Acceptance n (%)***
Addition of a drug	11 (1.9%)	7 (63.6%)	5 (1.1%)	3 (60.0%)
Drug dose level adjustment	62 (910.4%)	35 (56.5%)	131 (27.5%)	87 (66.4%)
Optimization of administration	6 (1.0%)	5 (83.3%)	16 (3.4%)	8 (50.0%)
Definitive discontinuation of a drug	281 (47.3%)	229 (81.5%)	58 (12.2%)	44 (75.9%)
Drug substitution/switching	48 (8.0%)	34 (70.8%)	165 (34.7%)	86 (52.1%)
Treatment monitoring	78 (13.1%)	63 (80.8%)	24 (5.0%)	17 (70.8%)
Missing data	108	50	77	31

Pharm.: pharmaceutically

* The proportion was calculated by dividing the number by the total number of pharmaceutically relevant alerts for each type of action.
